# Metabolomic Insight into Donation After Circulatory-Death Kidney Grafts in Porcine Autotransplant Model: Normothermic Ex Vivo Kidney Perfusion Compared with Hypothermic Machine Perfusion and Static Cold Storage

**DOI:** 10.3390/ijms26136295

**Published:** 2025-06-30

**Authors:** Iga Stryjak, Natalia Warmuzińska, Kamil Łuczykowski, Kacper Wnuk, Hernando Rosales-Solano, Patrycja Janiszek, Peter Urbanellis, Katarzyna Buszko, Janusz Pawliszyn, Markus Selzner, Barbara Bojko

**Affiliations:** 1 Department of Pharmacodynamics and Molecular Pharmacology, Faculty of Pharmacy, Nicolaus Copernicus University in Torun, Collegium Medicum in Bydgoszcz, 85-089 Bydgoszcz, Poland; 2 Department of Biostatistics and Biomedical Systems Theory, Faculty of Pharmacy, Nicolaus Copernicus University in Torun, Collegium Medicum in Bydgoszcz, 85-089 Bydgoszcz, Poland; 3 Department of Chemistry, University of Waterloo, Waterloo, ON N2L 3G1, Canada; 4 Ajmera Transplant Center, Department of Surgery, Toronto General Hospital, University Health Network, Toronto, ON M5G 2N2, Canada; 5 Department of Medicine, Toronto General Hospital, Toronto, ON M5G 2C4, Canada

**Keywords:** kidney transplantation, metabolomics, graft quality assessment, kidney perfusion, solid-phase microextraction SPME, liquid chromatography–mass spectrometry, LC–MS

## Abstract

Organ shortage is a major challenge in transplantation, prompting the use of extended criteria donor grafts. These require improved preservation techniques and reliable methods to assess graft function. This study aimed to evaluate changes in the kidney metabolome following three preservation methods: normothermic ex vivo kidney perfusion (NEVKP), hypothermic machine perfusion (HMP) and static cold storage (SCS) in porcine autotransplant models. A chemical biopsy allowed minimally invasive sampling of metabolites, which were analyzed using liquid chromatography coupled with high-resolution mass spectrometry. The results highlighted metabolites affected by ischemia and oxidative stress in donor kidneys, as well as changes specific to each preservation method. Differences were observed immediately after transplantation and reperfusion and several days post-surgery. NEVKP was associated with the activation of physiological anti-oxidative and anti-inflammatory mechanisms, suggesting potential protective effects. However, some metabolites had dual roles, which may influence future graft treatment designs. HMP and SCS, while reducing energy demand in cells, also limit physiological repair mechanisms. These findings provide a basis for improving graft assessment and organ preservation, with chemical biopsy serving as both a tool for discovery and a potential diagnostic method for monitoring graft quality.

## 1. Introduction

Renal replacement therapy (RRT) is a term used to describe forms of treatment such as kidney transplantation and dialysis (hemodialysis or peritoneal dialysis) that replace the function of the kidneys and sustain the life of patients with kidney failure. Each of these methods have both advantages and disadvantages, but taking into account the quality of life, mortality, survival and morbidity, kidney transplantation is the best choice. Moreover, dialysis is by far more expensive than renal transplant over the mean patient lifespan [1,2,3]. However, the demand for kidney grafts is much larger than their supply. This situation forces an increased access to renal transplants. Therefore, to increase the number of transplants performed, a program enabling the use of ECD kidneys was implemented. Expanded criteria donors (ECD) are those (patients/persons) over the age of 60 or 50–59 years of age with at least two of the following: terminal serum creatinine > 1.5 mg/dL, history of hypertension, or cerebrovascular as the cause of death [4,5,6,7]. The percentage of deceased kidney donors with expanded criteria increased about 30% in Europe in the recent years [4]. Obviously, the risk of ECD graft loss is greater than with transplants from standard criteria donors (SCDs), but kidney ECD transplantation offers more benefits such as higher survival rate and a better quality of life in comparison to patients treated with dialysis, especially in a group of patients older than 70 years old [4,5]. The 2019 Organ Procurement and Transplantation Network/Scientific Registry of Transplant Recipients (OPTN/SRTR) Annual Kidney Data Report emphasized that slightly above 40% of patients on a waiting list (average for all ages) is willing to accept the ECD or Kidney Donor Profile Index (KDPI) > 85% organs. For the 65 years old patients and above it is even >60% [8]. Therefore, to maintain or increase the performance of grafts in the period of preservation, the hypothermic machine perfusion of organs became widely applied. Hypothermia helps to slow down the metabolism and, consequently, the demand for energy and oxygen. At the same time, perfusion sustains mechanical homeostasis to vascular endothelium. However, a sudden increase in temperature after revascularization and reperfusion may lead to “rewarming injury” involving mitochondrial respiratory dysfunction and, consequently, apoptosis. To address this issue, normothermic machine preservation was proposed. The reports demonstrated the superior performance of the technique over static and dynamic hypothermic preservations [9,10,11,12]. Despite that, limited studies provide insight into the biochemical alteration associated with particular strategies.

Undoubtedly, one of the fundamental problems of current transplantation is the objective assessment of organ quality before transplantation. The basic stage in deciding whether a kidney will be accepted for transplantation or rejected is its visual appraisal by the transplant team [13]. Thanks to the macroscopic examination of the kidneys, we can find vascular and anatomical changes or damages, thrombosis, atherosclerosis, infarction, fibrosis and scarring and a kidney tumor [14]. Unfortunately, the method is not objective and depends mainly on the experience of the surgeon [13,14,15]. Therefore, it is important to develop a reliable assessment of the actual quality of organs. Metabolomics is a scientific concept of studying an entire set of small molecules, called a “metabolome”, within the cell, tissue, or organism. Metabolome is sensitive to genetic modification, physiological and environmental stimuli as well as to the altered kinetic activity of enzymes, which makes it possible to observe changes in biochemical pathways. Implementing metabolomics to transplantation enables the monitoring of donor organ viability or the detection of organ rejection [16,17]. Among the most recent advances in this area is the integration of multi-omics data with clinical information to better interpret the results for predicting allograft rejection [18]. The authors even use the term “transplantomics” for the mentioned approach. Shen et al. proposed to implement model-informed precision dosing (MIPD) using pharmacogenomics and pharmacometabolomics to support therapeutic drug monitoring of sacrolimus in children, as the current strategy utilizing TDM alone results in ineffective treatment, variable pharmacokinetics and toxicity [19]. Characterization of potential biomarkers for the assessment of graft quality before transplantation and evaluation of graft-related outcomes is considered the mainstream of the current metabolomic studies in the review by Kvietkauskas et al. [17]. The authors draw attention to the utilization of the samples, which can be collected non-invasively. The analysis is usually performed using biological fluids because of the invasiveness of tissue biopsy and its potential complications. Solid-phase microextraction method (SPME) is a modern, versatile analytical method of sample preparation that combines (integrates) sample collection, metabolism inhibition and compound extraction into a single step, which enables rapid analysis on-site [20,21,22]. The most important advantages of the method are its simplicity, speed of analysis and the use of small amounts of organic solvents and test samples. In addition, the sample preparation method is cost-effective and offers high throughput. Also, the technique allows the determination of unstable, short-lived metabolites [20,21,22]. SPME, in contrast to other commonly used methods for metabolite and biomarker determination, is a safe and low-invasive tool for tissue analysis that enables simultaneous research in different regions of the organ. It is possible thanks to the use of a microprobe with a biocompatible extraction phase, which is inserted directly in the examined organ with negligible damage. Combining this minimally invasive chemical biopsy tool with analysis of the global metabolome and lipidome offers the unique opportunity to monitor temporally and spatially resolved changes in the graft in a peritransplant period [23,24,25].

The current work aimed to reveal metabolomic changes occurring in kidney grafts subjected to three preservation methods: static cold storage (SCS), hypothermic machine perfusion (HMP) and normothermic ex vivo kidney perfusion (NEVKP). The autotransplant porcine model allowed for the controlled experimental conditions. Utilization of SPME microprobes enabled monitoring of the alterations in the donor before transplantation, during warm ischemia, over the preservation period, following the transplantation and reperfusion and on the third post-transplant day.

## 2. Results and Discussion

The proposed method was employed to investigate changes in the metabolomic profiles during kidney preservation using SCS, HMP and NEVKP. At first, principal component analysis (PCA) was used to confirm the quality of the instrumental analysis. As shown in Figure S1, pooled QC samples formed a tight cluster, confirming the results’ quality. Analysis of the results was divided into four parts: (i) analysis of the influence of warm ischemia on metabolomic profiles of kidney, (ii) comparison of three types of organ preservation, (iii) monitoring changes across time of preservation and (iv) investigation of the influence of the overall transplantation procedure on kidney grafts.

### 2.1. Influence of Warm Ischemia on Kidney Metabolomic Profiles

**Figure 1:**
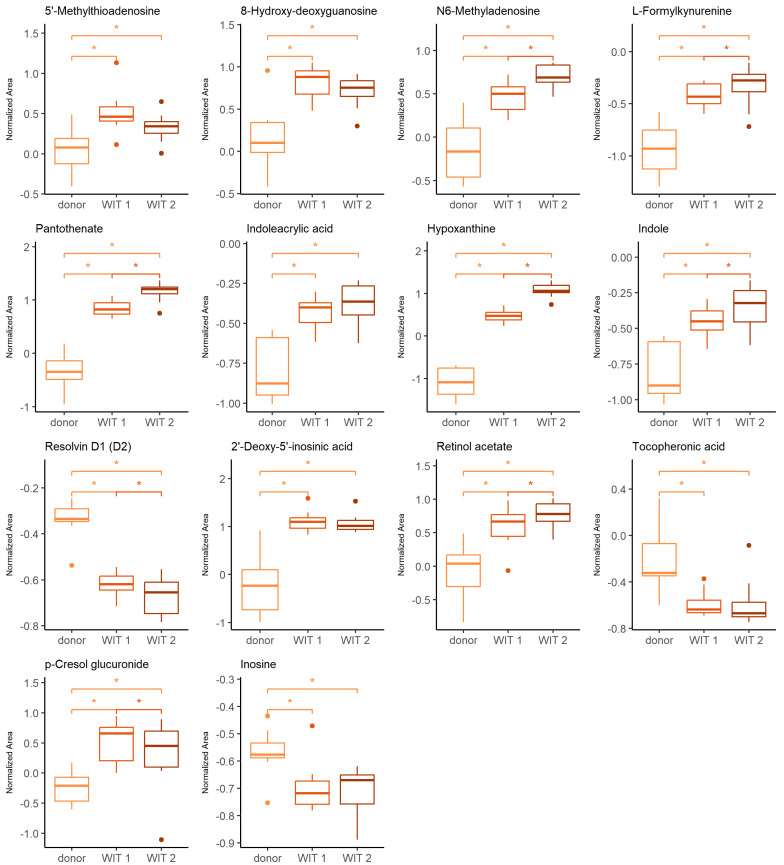
Change in levels of the selected metabolites during warm ischemia. Boxplots display normalized peak areas for both positive- and negative-mode analyzes. The rectangle’s height represents the normalized peak areas in the interquartile range (Q1 and Q3). Upper whisker—the largest data point excluding any outliers. Lower whisker—the lowest data point excluding any outliers. The median normalized peak area of each group is indicated with a square. Dots as observations >1.5 IQR (interquartile range) from the rectangle. Color legend: orange—donor, light red—WIT 1, dark red—WIT 2. The asterisk represents significant difference between the groups (*p* < 0.05).

The Friedman test was used to identify changes that occurred during warm ischemia. Discriminative changes were found mainly between the donor and the first time point of warm ischemia time (WIT). The compounds belong mainly to the groups of amino acids and derivatives, nucleosides and purine derivatives. The influence of ischemia and oxidative stress was already widely investigated in the context of graft preservation; therefore, the current manuscript will focus mainly on the differences occurring after the WIT, when the kidneys are subjected to HMP, NEVKP and SCS. However, a few findings, of which the vast majority is discussed later in the article, during a comparison of the aforementioned preservation techniques, are also highlighted here. The boxplots of selected statistically significant metabolites are shown in Figure 1.

The warm ischemia significantly influenced the levels of metabolites involved in purine and pyrimidine metabolism and amino acid metabolism, particularly tryptophan, arginine and proline and cysteine and methionine, as well as phenylalanine. The dysregulation of amino acid metabolism in renal injury, particularly ischemia-reperfusion injury (IRI) was widely reported [26,27,28,29,30]. It was postulated that amino acid metabolism may be a significant pathway in IR-AKI development [27]. The evidence of a significant effect of oxidative stress during the WIT is an increasing level of 8-hydroxy-deoxyguanosine, a marker of oxidative DNA [31]. Several metabolites found in the current studies and showing statistical significance in differentiating subsequent groups are products of epigenetic modifications. Prolonged WIT corresponded to the significant increase of N6-methyladenosine (m6A) and 2′-deoxy-5′-inosinic acid (Figure 1). In general, the epigenetic regulation includes DNA methylation and histone and non-coding RNA modifications leading to an alteration in gene expression without a change in DNA sequence [32,33]. In recent years, there has been a growing interest in the role of these factors in the development of complications related to kidney transplantation, including IRI, immune response or development of interstitial fibrosis/tubular atrophy [32,33,34,35]. Investigation of particular modifications requires different analytical techniques, including liquid chromatography coupled to mass spectrometry. Individual modified nucleosides or nucleobases are degradation products of the nucleic acids, and they can be analyzed in a targeted manner or as a part of the global metabolomics analysis. Because of the high dynamic of the RNA modification process and the instability of this nucleic acid, the characteristic methylation products of RNA, namely N6-methyladenosine, N1-methyladenosine, 5-methylcytosine and 7-methylguanosine, gained particular attention. However, it needs to be emphasized that the number of various RNA modifications identified to date reached 170 [33]. Both N6-methyladenosine (m6A) and 2′-deoxy-5′-inosinic acid were also reported as compounds discriminating hypothermic- and normothermic-preserved grafts. Also, hypoxanthine, pantothenate, indole, indoleacrylic acid, L-formylkynureine, tocopheronic acid, p-cresole glucuronide and resolvin D are examples of metabolites, which levels were also altered during kidney preservation. The relevant discussion to the changes of these compounds is presented in the following sections.

### 2.2. Comparison of Different Types of Kidney Preservation Methods

**Figure 2:**
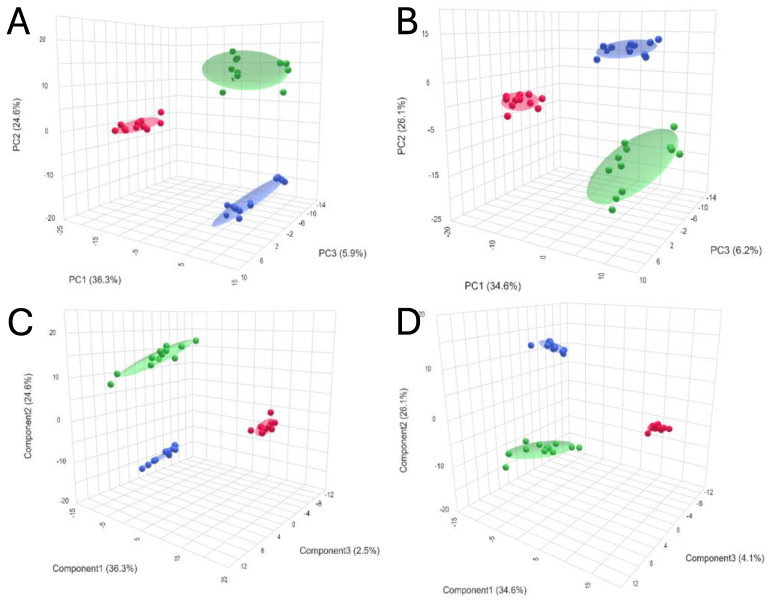
3D score plots of PCA and PLS-DA showing separation between different types of kidney preservation in positive ((**A**)—PCA; (**C**)—PLS-DA) and negative ((**B**)—PCA; (**D**)—PLS-DA) ionization modes. Color legend: HMP—red; NEVKP—green; SCS—blue.

Chemometrics analysis was performed to visualize the data and investigate differences in kidney metabolomic profiles between the SCS, NEVKP and HMP groups and to identify potential outliers (none were found). The two-dimensional scoring plots (PC1 vs. PC2) presented in Figure 2A,B revealed differences in the patterns of samples harvested during different types of preservation. A clear clustering of the samples obtained after subjecting kidney grafts to all three preservation methods was observed. Furthermore, the PLS-DA method was used to more accurately model differences in profiles between different types of kidney preservation (Figure 2C,D). Each model was validated with leave-one-out cross-validation.

**Figure 3:**
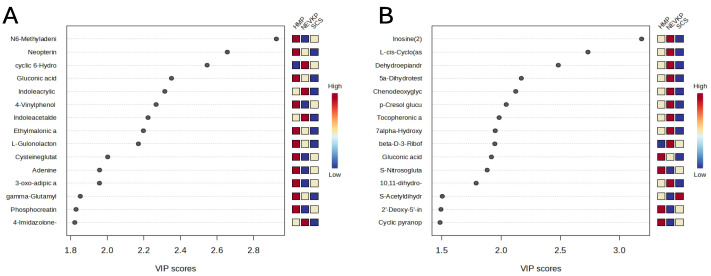
The VIP plot of the top 15 metabolites differentiating HMP, NEVKP and SCS during the preservation of kidney grafts. Results for positive (**A**) and negative (**B**) ionization modes.

**Figure 4:**
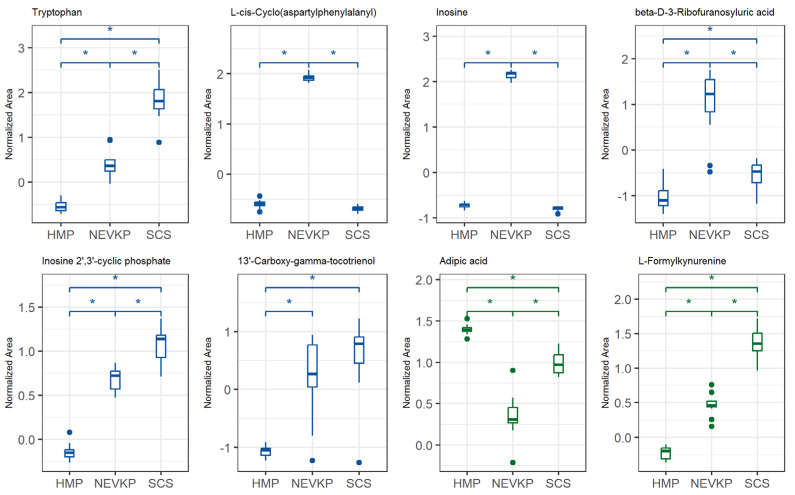
Differences in levels of the selected metabolites (FDR-adjusted *p*-value < 0.05) between three types of kidney preservation. Boxplots display normalized peak areas for both positive (green)- and negative (blue)- mode analyzes. The rectangle’s height represents the normalized peak areas in the interquartile range (Q1 and Q3). Upper whisker—the largest data point excluding any outliers. Lower whisker—the lowest data point excluding any outliers. The median normalized peak area of each group is indicated with a horizontal line. The asterisk represents significant difference between the groups (*p* < 0.05).

This statistical analysis yields a set of compounds that differentiate the types of kidney preservation. A VIP score value > 1 was selected as a cut-off value. The VIP plots of the top 15 metabolites differentiating HMP, NEVKP and SCS during the preservation of kidney grafts are presented in Figure 3. In addition, the Kruskal–Wallis test was used to compare the NEVKP, HMP and SCS groups. Boxplots of the selected metabolites which maintained statistical significance after applying FDR correction are shown in Figure 4.

The analysis of the levels of the metabolites enabled us to find evidence that the conditions of HMP and NEVKP activate different mechanisms mitigating ischemia and oxidative stress and their consequences. Still, many compounds indicate that normothermic machine perfusion overperforms hypothermic preservation. The number of compounds, mainly upregulated in hypothermic methods or downregulated in NEVKP, indicates intensified effects of cellular alterations and tissue injury caused by ischemia and oxidative stress in the former strategies. Contrarily, the metabolites upregulated in NEVKP indicate their protective role in ischemia and are mainly related to maintained enzymatic activity, which enables the activation of physiological mechanisms in the stored grafts.

A pronounced change was observed in N6-methyladenine and 2′-deoxy-5′-inosinic acid (deoxyinosine monophosphate, dIMP), which are linked to epigenetic modifications and WIT as mentioned earlier in this work. dIMP, a key purine synthesis intermediate, was elevated in hypothermic kidney preservation. It can convert to deoxyadenosine monophosphate (dAMP) and deoxyguanosine monophosphate (dGMP), building blocks for DNA synthesis, but oxidative stress may cause the accumulation of deaminated purines, leading to potential DNA mutations. Behmanesh et al. demonstrated that the inactivation of inosine triphosphatase (ITPA), an enzyme hydrolyzing dITP to dATP, may lead to the accumulation of free dITP [36]. The low level of dIMP in the NEVKP graft observed in our studies compared to hypothermic-preserved kidneys may be evidence that maintaining a more physiological environment of organs enables maintaining repair mechanisms and reducing negative effects of oxidative stress and ischemia. On the other hand, inosine was upregulated in NEVKP and downregulated in SCS. Inosine supports energy production under glucose deficiency and may reduce inflammation and promote cell survival during stress [37]. Modis et al. stated the relevancy of testing inosine for protection against various forms of warm and cold liver ischemia [38].

Another three compounds showing lower levels in NEVKP, phosphocreatine (PCr), oxoadipic acid and S-acetyldihydrolipoamide, are also related to energy production. PCr supports ATP regeneration during ischemia and acts as a spatial and temporal buffer for energy transfer within cells [39,40,41]. On the other hand, Maqdasy et al. evidenced that decreased phosphocreatine metabolism in white adipose tissue promotes pro-inflammatory responses [40]. We hypothesized that the low level of PCr in NEVKP corresponds to a maintained energy demand and restoration of the ATP pool, while a higher PCr level in hypothermic preservation is a consequence of lower energy demand, but it may also contribute to the development of inflammation. However, this concept requires further research. Limited data exist on oxoadipic acid and S-acetyldihydrolipoamide in ischemia, but oxoadipic acid has been noted as an early biomarker of myocardial infarction [42], while potential support of mitochondrial activity by acetyldihydrolipoamide-E, a specific form of S-acetyldihydrolipoamide, was shown in cancer cells [43].

An elevated level of cysteine-glutathione disulfide (CySSG) in hypothermic-preserved grafts compared with NEVKP was also noted in the current study. Hendriks et al. demonstrated that lowering the temperature indeed dramatically decreases mitochondrial oxygen consumption, but it also induces failure of endogenous antioxidant capacity [44]. Jones et al. stated that the major cellular thiol/disulfide systems are not in redox equilibrium and respond differently to chemical toxicants and physiologic stimuli [45]. This would explain the observed phenomenon of the upregulation of the CySSG level in hypothermic grafts, which are at a greater risk of oxidative stress. Gamma-glutamylglycine (γ-GG), a product of glutathione breakdown by gamma-glutamyl transferase (GGT), was elevated in HMP kidneys indicating oxidative stress [46], but also supporting glutathione recycling to sustain antioxidant defense [47]. Our results also showed an alteration of S-nitrosoglutathione (GSNO) that serves as a reservoir for nitric oxide (NO) and plays a significant role in various physiological and pathological processes, including those related to ischemic injury. Interestingly, the evidence suggests that specific changes in GSNO levels can depend on various factors, including the duration and severity of ischemia and the tissue type involved. Liu et al. found that GSNO administration could provide protective effects against myocardial IRI in non-diabetic mice, suggesting that GSNO levels may rise as a compensatory response to enhance NO signaling and mitigate oxidative damage. At the same time, they noted aggravated injury in diabetic mice [48]. The authors concluded that diabetes may cause superoxide overproduction, increase NO inactivation and peroxynitrite formation and thus convert GSNO from a cardioprotective molecule to a cardiotoxic molecule. In the context of ischemic stress and renal injury, a study by Fan et al. indicated that GSNO improved outcomes after ischemic events in a septic acute kidney injury model via mechanisms related to inhibition of inflammation, oxidation and apoptosis [49].

HMP kidneys showed higher levels of L-gulonolactone, a precursor of ascorbic acid, suggesting enhanced antioxidant defense during ischemia [50]. Among the compounds which were upregulated in the NEVKP grafts in the current study was cyclic 6-hydroxymelatonin. The cyclic form of 6-hydroxymelatonin is part of the broader metabolic pathway of melatonin, present mainly in the liver and kidneys [51]. Melatonin and its cyclic and hydroxy derivatives exert antioxidant properties. For instance, the protective effects of melatonin against renal IRI have been documented, with studies indicating that melatonin administration can reduce markers of oxidative stress and improve renal function following ischemic events [52]. It also influences NO production, which plays a dual role in ischemic injury. While NO can have protective effects, excessive production, especially from inducible nitric oxide synthase (iNOS) during ischemia, can lead to further oxidative damage. Melatonin and its metabolites may help modulate NO levels, potentially reducing the harmful effects of excessive NO while preserving its beneficial actions [53]. Research has indicated that cyclic derivatives of melatonin exhibit significant biological activity, including antioxidant properties [54].

The current study also evidenced the involvement of indole derivatives in the graft preservations. The indoleacrylic acid (IAA) was previously reported to affect the level of unsaturated fatty acids in the membrane by regulating cell lysophospholipase activity and significant changes in phospholipids [55]. The authors postulated that low IAA levels may indicate cellular membrane permeability damage, while stimulation of IAA production can promote an anti-inflammatory response. Although these observations were applied to cancer, the described dysregulation of IAA corresponds to our study, where downregulation of the metabolite was noted in SCS and upregulation in NEKLP. A similar profile applies to indole acetaldehyde. This can be explained by the study by Huang et al. who reported a decreased level of indole acetaldehyde in hyperuricemia rats compared with that in normal rats [56]. They highlighted the protective and anti-inflammatory roles of the compound, suggesting that it may play a beneficial role in conditions associated with tissue injury.

Several metabolites were upregulated in NEVKP compared to hypothermic preservation, including DHEAS, 5α-dihydrotestosterone sulfate and others. DHEAS has been linked to protection against ischemic injury [57], with low levels associated with higher stroke risk [58]. Its elevated presence in NEVKP may indicate better kidney protection, while low levels in SCS suggest higher vulnerability. Though not directly studied in kidney preservation, DHEA (the precursor of DHEAS) has shown protective effects against oxidative stress and renal injury [59]. The authors hypothesized that the protective effects of DHEA against oxidative injury are attributed to its ability to integrate into cell membranes, thereby enhancing membrane stability and resistance to oxidative stress [59]. Similarly, while data on 5α-dihydrotestosterone sulfate are limited, androgens like 5α-dihydrotestosterone (DHT) are known to influence inflammation and oxidative stress [60].

Our study showed elevated p-cresol levels in NEVKP compared to hypothermic methods (Figure 4). p-Cresol glucuronide is formed via UDP-glucuronosyltransferase (UGT) enzymes in various tissues beyond the liver, including kidneys [61]. It is less active under hypothermia, explaining lower levels in cold conditions. While glucuronidation aids detoxification, accumulation of p-cresol and its conjugates can trigger oxidative stress and inflammation, contributing to tissue injury. These metabolites have been associated with cardiovascular disease and endothelial dysfunction in patients with chronic kidney disease (CKD) [62]. They can impair endothelial cell proliferation and alter inflammatory responses and the production of inflammatory cytokines and affect cell cycle regulation [63,64]. Free p-cresol may also cause endothelial microparticle shedding, worsening dysfunction in patients with end-stage renal failure [65].

Tocopheronic acid (α-tocopherol), a lipid-soluble antioxidant, was found at the highest levels in NEVKP and lowest in SCS kidneys. It protects cells by neutralizing ROS and preventing lipid peroxidation. Although it is consumed during this process, it can be regenerated by other antioxidants, preserving its activity [66]. The elevated levels in NEVKP suggest more effective regeneration, supporting sustained antioxidant defense.

Current studies revealed the downregulation of 7alpha-Hydroxy-3-oxo-4-cholestenoate (7-HOCA) in SCS grafts. It is formed from 27-hydroxycholesterol (27-OHC) via CYP7B1, but ischemia may hinder this conversion due to reduced enzyme activity and clearance, leading to 27-OHC buildup [67]. Heverin et al. reported that the conversion of 27-OHC to 7-HOCA might help modulate inflammation in the brain, as 7-HOCA is less cytotoxic than its precursor, 27-OHC [68]. This would support the hypothesis that the highest level of 7-HOCA in NEVKP seen in our studies is due to the maintained enzymatic activity. Consequently, normothermic machine perfusion enables utilizing physiological mechanisms of tissue protection.

Another metabolite that exhibited a higher level in NEVKP compared to HMP and SCS is beta-D-3-ribofuranosyluric acid (urate-3-ribonucleoside). The reports on the role of this particular compound in human health are very limited. The study by Zhang et al. identifies urate-3-ribonucleoside among other metabolites that showed a recovery tendency toward the normal level in patients with ovarian cancer after surgery [69]. No hypothesis related to this change was put forward due to the small size of the studied group. However, urate and its derivatives were reported as compounds possessing protective functions and mitigating oxidative stress. Therefore, the high level of urate-3-ribonucleoside in our study might be linked to the better defensive potential of grafts undergoing normothermic perfusion. Still, further studies are needed to support this theory.

Surprisingly, the current study showed an increased level of 10,11-dihydro-20-dihydroxy-LTB4 in NEVKP grafts and a decrease in SCS. LTB4 and its derivatives are potent pro-inflammatory mediators; therefore the mentioned profile of 10,11-dihydro-20-dihydroxy-LTB4 across the preserved grafts requires further investigations.

### 2.3. Changes Across Time

**Figure 5:**
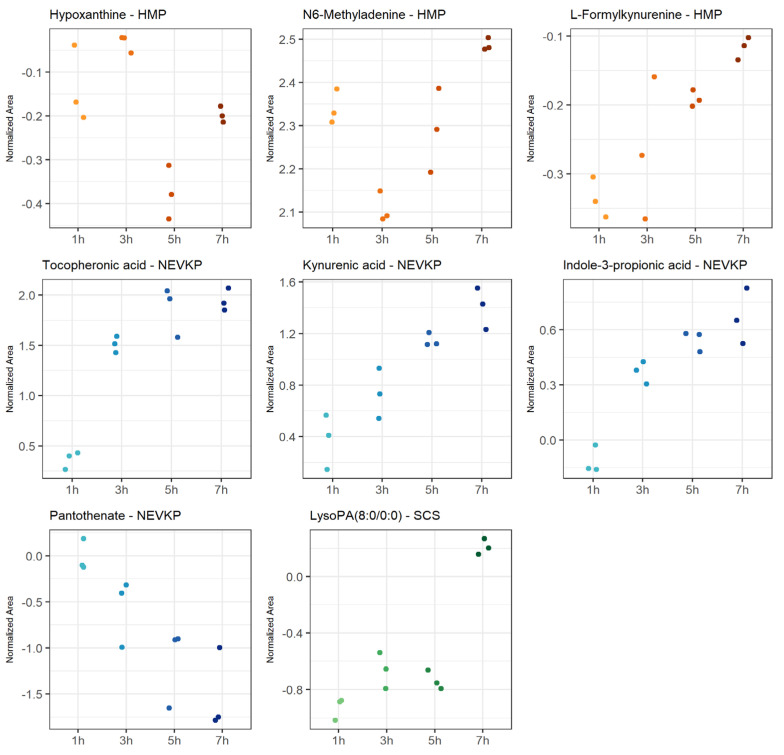
Strip plots of selected metabolites showing statistically significant correlation in changes throughout kidney preservation. Blue—NEVKP; red/orange—HMP; green—SCS.

The most statistically significant (*p* < 0.05 after FDR correction) changes over time detected in the negative ionization mode were observed for HMP, and significantly fewer for NEVKP and SCS. In the case of positive ionization, there were fewer metabolites changing over time, but similarly to negative ionization, the most was observed for HMP, and fewer for normothermic and SCS conditions. The number of compounds showed a linear trend, particularly for the mechanic perfusions. Strip plots of selected compounds that demonstrated significant alterations during preservation are shown in Figure 5, and detailed information about the correlation coefficient and raw *p*-value are shown in Table S1.

The machine perfusion induced dynamic changes during organ preservation compared to static preservation. It was observed that, in machine perfusion, many compounds are related to tryptophan metabolism. In particular, metabolites involved in the kynurenine pathway indicated activation of the protective mechanisms in HMP and NEVKP. Some studies demonstrated a protective role of the kynurenine pathway in cardiac ischemia [70]. However, in reviewing a kynurenic pathway as a potential therapeutic target in kidney transplantation, Zakrocka et al. emphasized that indoleamine 2,3-dioxygenase (IDO) activity, the enzyme determining activation of the entire kynurenine pathway, is highly dependent on the balance between pro- and anti-inflammatory conditions. Moreover, the final effect of the kynurenic pathway on cellular survival and immune processes depends on the multiple and often opposing effects of its individual metabolites [71]. It is worth mentioning that the kynurenine serum level was postulated as a reliable diagnostic tool for the assessment of post-transplant inflammatory complications in an early stage and for monitoring the efficacy of therapeutic interventions [72]. Further, NEVKP was associated with the liberation of several different antioxidants, e.g., pantothenic acid and the aforementioned tocopheronic acid. The former has a protective role in ischemia, mitigating oxidative stress and lipid peroxidation by increasing reduced glutathione (GSH), co-enzyme A (CoA) and intracellular adenosine triphosphate (ATP) synthesis [73]. Pantothenate was also reported as one of the candidates for biomarkers of monitoring kidney function in post-transplant patients [74]. In HMP grafts, among the compounds exhibiting significant alterations during the 8 h perfusion was hypoxanthine. The metabolite is considered a graft ischemia marker as during renal ischemia; ATP degradation results in the production of hypoxanthine, which is further metabolized by xanthine oxidase during reperfusion into xanthine with concomitant liberation of cytotoxic oxygen free radicals, leading to renal injury [75]. However, Wijermars et al. stated that the hypoxanthine–xanthine oxidase axis is not involved in the initial phase of clinical-transplantation-related IRI [76]. N6-methyladenine, also reported in the current study as one of the metabolites altered by warm ischemia, was significantly changed during the HMP course. On the other hand, the preservation of grafts in static hypothermia resulted in the significant alteration of lysophosphatidic acids (LPA). This bioactive lipid is known for its involvement in the development of renal fibrosis, which is often the cause of late graft loss [77].

### 2.4. Influence of Transplantation Procedure on Kidney Grafts

A fold change analysis with a Welch’s *t*-test with FDR correction was used to evaluate the influence of the overall transplantation procedures (from donor to reperfusion and postoperative day 3). A comparison of the results obtained for donor and immediate reperfusion sampling points revealed that, for both perfusion methods, the number of statistically significant downregulated metabolites was higher than for SCS. Interestingly, with regards to upregulated metabolites the numbers were lower and similar for all three. Analysis of the results for the POD3 showed that the numbers of down- and upregulated metabolites for NEVKP dropped, compared to immediate reperfusion data, while for SCS it remained at about the same level as it was noted after revascularization. Table S2 represents a detailed list of the results.

Among the metabolites which changed significantly when comparing pre-transplant and post-transplant grafts were compounds already described in this study, e.g., N6-methyladenine, indoleacrylic acid and metabolites of kynurenine pathway. In addition, metabolites which did not show significant alterations during preservation were noted. In NEVKP, aspartic acid exhibited an increasing trend. The compound was tested for its potential role in attenuating oxidative stress damages induced by ischemia/reoxygenation via facilitating NAD regeneration from NADH [78]. Another metabolite elevated not only in NEVKP, but also in HMP, is reduced nicotinamide riboside (1-(beta-D-Ribofuranosyl)-1,4-dihydronicotinamide). Zhao et al. demonstrated that supplementation of nicotinamide riboside (NR), a precursor to NAD+ engaged in cellular function and energy homeostasis, mitigates mitochondrial damage and inhibited apoptosis during myocardial IRI [79]. Earlier, Toropova et al. reported that NR can be used to reduce the IRI of small intestines and to preserve intestinal grafts until transplant [80]. It was also observed in the current study that levels of retinoids were significantly increased in both perfusion methods. Pretreatment with retinoic acid was reported in several studies as an effective way of ameliorating IRI [81,82]. An upregulation of metabolites of the retinol metabolism pathway in the heart which then prolonged ex situ heart perfusion was reported by Olkowicz et al. [25]. The same study showed alterations of the D-series resolvins, and significant upregulation was observed in the current studies in NEVKP- and HMP-graft post-reperfusion. It was previously demonstrated that the D-series resolvins can significantly reduce neutrophil infiltration and protect organs during acute organ IRI [83]. Moreover, Kasuga et al. emphasized that these resolvins act locally on target cells and can be metabolically inactivated, which underscores the importance of their timely action in the inflammatory milieu [84]. The noticeable changes occurring after reperfusion in HMP-preserved grafts were also in the levels of purines and purine phosphates, bile acids and phospholipids. While the roles of the first two groups were mentioned earlier in this work, the lipidomics analysis in the studied cohorts was described in detail elsewhere [23]. The SCS-preserved grafts characterized less dynamic change compared to machine perfusion strategies, but among the significantly altered compounds were those indicating developing inflammation and tissue damage, e.g., prostaglandins and leukotrienes, as well as isoxanthopterin contributing to endothelial dysfunction [25].

## 3. Materials and Methods

### 3.1. Chemicals

Pierce LTQ Velos ESI Positive External Calibrant Solution and Negative Ion Calibration Solution were purchased from Anchem (Anchem, Warsaw, Poland). Water (LC/MS Optima grade) and formic acid were purchased from Merck (Merck, Poznań, Poland), and acetonitrile and methanol (all LC/MS Optima grade) were purchased form Alchem (Alchem, Torun, Poland). Prototypes of biocompatible SPME mixed-mode probes were kindly provided by Supelco (Bellefonte, PA, USA). The microprobes, also called SPME fibers, were made of a thin wire (nickel-titanium alloy of 0.2 mm in diameter) with a 7 mm tip coated with a mixed-mode MM extraction phase (C18 and benzenesulfonic acid).

### 3.2. Animal

Eight three-month-old, male Yorkshire pigs (ca. 30 kg) were housed for 1 week before the experiments. Water and food were provided ad libitum. All animal procedures and care were regulated according to the Principles of Laboratory Animal Care by the National Society for Medical Research and the Guide for the Care of Laboratory Animals published by the National Institutes of Health. Per the “Ethics Statement and Patient Consent”, the study protocol was approved by the Animal Care Committee of the Toronto General Research Institute, Ontario, Canada (AUP # 3652.16 and AUP # 3652.19).

### 3.3. Study Design

**Figure 6:**
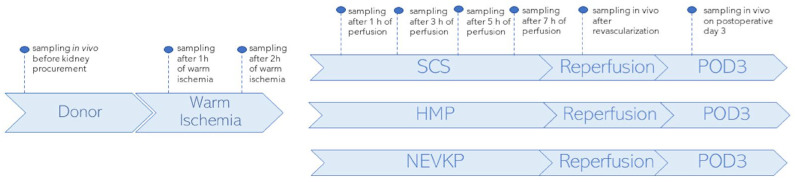
Study design. SPME sampling was performed in vivo prior to kidney procurement; after 1 h and 2 h of warm ischemia; after 1 h, 3 h, 5 h and 7 h of perfusion; in vivo immediately after revascularization (reperfusion) and in vivo under deep anesthesia at the time of sacrifice on postoperative day 3 (POD3). Three types of kidney preservation methods—SCS, HMP and NEVKP—were compared in the DCD porcine model of renal autotransplantation. Reprinted with permission from [23].

A porcine autotransplantation model of renal donation after circulatory death (DCD) was used in the study. It aimed to compare metabolomic changes occurring in kidney grafts when subjected to three preservation methods: SCS (n = 3), HMP (n = 2) and NEVKP (n = 3). For all three methods, organ preservation lasted 8 h. The autotransplantation and anesthetic procedures, warm ischemia induction and NEVKP, HMP and SCS conditions are described elsewhere [85]. The kidney sampling was performed by direct introduction of the microextraction probes into intact organs. The SPME sampling was performed in vivo before retrieval; after 1 h and 2 h of warm ischemia; after 1 h, 3 h, 5 h and 7 h of preservation; again in vivo immediately after revascularization (reperf); and in vivo under deep anesthesia at the time of sacrifice on postoperative day 3 (POD3). The study protocol was already described for the lipidomics analysis of the studied cohort, and it is shown in Figure 6.

Prior to the experiments, all probes were kept in a methanol/water (50:50 *v*/*v*) solution for 60 min to activate the extraction phase. Subsequently, fibers were rinsed with purified water for a few seconds to remove the potential remains of the organic solvent. The extractions with SPME fibers were performed for 30 min at each time point from the kidney cortex. Following the sampling, the probes were retracted from the organ, quickly rinsed with water and then gently dried with wipes to remove potential tissue or blood residue loosely attached to the coating. After that, the fibers were secured in empty glass vials and stored at −80 °C until analysis. On the day of analysis, all the probes were desorbed in the glass inserts containing 200 μL of an acetonitrile:water (80:20 *v*/*v*) solution. Desorption was performed with agitation (at 1200 rpm using the BenchMixer™ MultiTube Vortexer from Benchmark Scientific, Edison, NJ, USA) for 120 min. Along with the probes used for the kidney samplings the extraction blanks were analyzed. The extraction blanks were prepared using probes undergoing the same protocol as the rest of the fibers (preconditioning, desorption, etc.), but they were not used for kidney sampling. The analysis of extraction blanks aimed to identify and remove from the data results any interferences and contaminants from the experimental environment.

### 3.4. Liquid Chromatography–High Resolution Mass Spectrometry Analysis (LC–HRMS)

Samples were analyzed using an LC–HRMS platform based on the coupling of a Dionex UltiMate 3000 RS autosampler, a Dionex Ultimate 3000 RS pump (Thermo Fisher Scientific, Dionex, Bremen, Germany) and a Q Exactive Focus high-resolution mass spectrometer (Thermo Fisher Scientific, Bremen, Germany). Data acquisition was performed using dedicated Thermo Scientific software, namely, Xcalibur 4.2 and Free Style 1.4 (Thermo Fisher Scientific, San Jose, CA, USA). The instrument was calibrated via external calibration every 72 h, resulting in a mass accuracy of <2 ppm. Within-sequence samples were randomized, and pooled quality control (QC) samples composed of 10 μL of each sample were run every 8–10 injections to monitor instrument performance.

Chromatographic separation was carried out in reversed-phase (RP) using a pentafluorophenyl column (Supelco Discovery HS F5, 2.1 mm × 100 mm and 3 μm) in positive and negative ionization modes. The mobile phases were prepared according to the method described previously in reference number [24]. The flow was set to 0.3 mL/min, and an injection volume of 10 μL was employed. The column temperature was set to 25 °C, and the sample vials were held at 4 °C in the autosampler. The mass spectrometer parameters in positive ionization mode were as follows: a sheath gas flow rate of 40 a.u.; an aux gas flow rate of 15 a.u.; a spray voltage of 1.5 kV; a capillary temp of 300 °C; an aux gas heater temp of 300 °C; an S-lens radio frequency level of 55%; an S-lens voltage of 25 V; and a skimmer voltage of 15 V. The scan range was set to *m*/*z* 80–1000 with a resolution of 70,000 full width at half maximum (FWHM). Acquisition was performed using an automatic gain control (AGC) target of 1 × 10^6^, with the C-trap injection time set to auto. In negative ionization mode, the mass spectrometer parameters were as follows: a sheath gas flow rate of 48 a.u.; an aux gas flow rate of 11 a.u.; a spray voltage of 2.5 kV; a capillary temp of 256 °C; an aux gas heater temp of 413 °C; an S-lens radio frequency level of 55%; an S-lens voltage of −25 V; and a skimmer voltage of −15 V. In this mode, the scan range was set to *m*/*z* 80–1000 with a resolution of 70,000 FWHM. Acquisition was performed using an automatic gain control (AGC) target of 1 × 10^6^, with the C-trap injection time set to auto.

### 3.5. Data Processing and Statistical Analysis

Metabolomic data processing was performed using the Compound Discoverer 3.1. (Thermo Fisher Scientific, San Jose, CA, USA) software with the following parameters: selected mass tolerance window of a max 3 ppm; peak intensity >100,000; signal-to-noise threshold >3; and a max sample-to-blank ratio > 5. The QC-based area was used for correction (min 80% coverage and max 30% RSD in QC). Features were putatively identified by searching for their exact molar weights in the CEU Mass Mediator with a 3 ppm mass tolerance. The peak areas for the obtained compounds were analyzed using MetaboAnalyst 6.0 and R Statistical Software (v. 4.4.2; R Core Team 2024). All of the missing values were replaced with small values assumed to be a detection limit. Data were normalized by median, log-transformation and Pareto scaling.

Principal component analysis (PCA) was used to evaluate the quality of the data. The Friedman test was used to evaluate the statistical significance of the metabolites expressing alterations during warm ischemia, and the Kruskal–Wallis test was used for differentiating the three studied preservation methods. For the Friedman test, the Conover post-hoc test with the Benjamini–Hochberg correction for multiple comparisons was chosen. For Kruskal–Wallis test, Dunn post-hoc test, also with the Benjamini–Hochberg correction was used. In addition, for the assessment of differences between SCS, HMP and NEVKP, principal component analysis (PCA) and partial least squares discriminant analysis (PLS-DA) were conducted to visually assess separation between sample groups, with variable importance in projection (VIP) scores >1 being considered significant. The PLS-DA model was 5-time cross-validated using leave-one-out cross validation. Two-dimensional score plots were generated to visually assess the separation of sample groups. To evaluate changes during kidney preservation, due to the small sample size at each time point (n = 3), one-way repeated measures analysis of variance (RM-ANOVA) with Greenhouse–Geisser correction was performed (only when the assumption of sphericity verified by Mauchly’s test was not met). Pearson’s correlation coefficient with FDR correction was used to identify metabolites with potential linear changes during preservation time. Finally, fold-change analysis with Welch’s *t*-test and FDR correction (using Benjamini–Hochberg procedure) was used to assess the influence of the overall transplantation procedures on the kidney metabolome.

## 4. Conclusions

The regulation of tissue response to ischemia, tissue injury and oxidative stress is very complex. The conditions associated with the given preservation method have a direct impact on the degree of these processes as they influence the activation of specific protecting or destructive mechanisms. Without any doubt, the temperature has the most pronounced effect on the preserved tissue, maintaining or inhibiting the metabolic activity of the graft. Hypothermia lowers energy demand in the cells, but it also restricts physiological repair mechanisms. On the other hand, oxygen and nutrient supply need to be adequate to the organ demand, which is the highest in normothermia. Based on the literature reports and our current findings, we may conclude that many compounds, which are known to have a protective role against ischemia and oxidative stress in certain conditions, exhibit Janus face. Therefore, it is very important to precisely determine and understand these relationships as the number of protective metabolites, particularly antioxidants, are considered supplements in preservation fluids or in donor pretreatment.

It is important to emphasize that, based on our best knowledge, this is the first report describing such alterations in the global metabolome directly in kidney grafts subjected to all three preservation methods, HMP, NEVKP and SCS, and showing changes over the entire procedures. Our previous studies, which utilized the same experimental strategy, demonstrated alterations at the lipidome level [23]. We may then postulate that the chemical biopsy can serve as a versatile sampling tool for discovery investigations, but it can also be translated into a diagnostic tool to monitor specific biomarkers of graft quality in the future.

It needs to be underlined that the current study has certain limitations; the studied cohort was small, and follow-up experiments are needed to confirm the potential of the given metabolites as future biomarkers. Also, the highest confirmation level of all compound identities should be performed prior to further targeted analysis. Nevertheless, the studies lie the ground for future experiments toward better graft assessment and improved organ preservation directly from the organs utilizing this non-destructive approach. With respect to the sampling strategy, which enabled the temporally resolved analysis, the new users should be aware that extraction relies on the equilibrium between the sample and the extractive phase; therefore, understanding the method fundamentals, particularly sampling at pre-equilibrium and its effect on reproducibility and sensitivity, is crucial to perform reliable experiments.

## Data Availability

The datasets generated during and/or analyzed during the current study are available from the corresponding author upon reasonable request.

## Supplementary Materials

The following supporting information can be downloaded at https://www.mdpi.com/article/10.3390/ijms26136295/s1.

## Abbreviations

The following abbreviations are used in this manuscript:

RRT	Renal replacement therapy
ECD	Expanded criteria donors
SCDs	Standard criteria donors
SPME	Solid-phase microextraction method
SCS	Static cold storage
HMP	Hypothermic machine perfusion
DCD	Donation after circulatory death
POD	Postoperative day
LC–HRMS	Liquid Chromatography–High Resolution Mass Spectrometry
PCA	Principal component analysis
PLS-DA	Partial least squares discriminant analysis
VIP	Variable importance in projection
RM-ANOVA	One-way repeated measures analysis of variance
WIT	Warm ischemia time
IQR	Interquartile range
IRI	Ischemia-reperfusion injury
AKI	Acute kidney injury
dIMP	Deoxyinosine monophosphate
dAMP	Deoxyadenosine monophosphate
dGMP	Deoxyguanosine monophosphate
ITPA	Inosine triphosphatase
PCr	Phosphocreatine
CK	Creatine kinase
AMI	Acute myocardial infarction
CySSG	Cysteine-glutathione disulfide
γ-GG	Gamma-glutamylglycine
GGT	Gamma-glutamyl transferase
ROC	Reactive oxygen species
GSNO	S-nitrosoglutathione
NO	Nitric oxide
iNOS	Inducible nitric oxide synthase
IAA	Indoleacrylic acid
DHEAS	Dehydroepiandrosterone sulfate
7-HOCA	7alpha-hydroxy-3-oxo-4-cholestenoate
DHT	Dihydrotestosterone
UGT	UDP-glucuronosyltransferase
CKD	Chronic kidney disease
7-HOCA	7alpha-Hydroxy-3-oxo-4-cholestenoate
27-OHC	27-hydroxycholesterol
IDO	Indoleamine 2,3-dioxygenase
GSH	Reduced glutathione
CoA	Co-enzyme A
ATP	Adenosine triphosphate
LPA	Lysophosphatidic acids
NR	Nicotinamide riboside
