# Supplementary material for: Metabolomic Insight into Donation After Circulatory-Death Kidney Grafts in Porcine Autotransplant Model: Normothermic Ex Vivo Kidney Perfusion Compared with Hypothermic Machine Perfusion and Static Cold Storage
Source: Int J Mol Sci. 2025 Jun 30;26(13):6295. doi: 10.3390/ijms26136295 (PMC12249885; doi:10.3390/ijms26136295)
Supplement: Supplementary file 1 [file ijms-26-06295-s001.zip › ijms-3689686-supplementary.pdf]

Supplementary Materials

Metabolomic Insight into Donation After Circulatory-Death Kidney Grafts in Porcine Autotransplant Model: Normothermic Ex Vivo Kidney Perfusion Compared with Hypothermic Machine Perfusion and Static Cold Storage

Iga Stryjak [1], Natalia Warmuzińska [1], Kamil Łuczykowski [1], Kacper Wnuk [2], Hernando Rosales-Solano [3], Patrycja Janiszek [1], Peter Urbanellis [4], Katarzyna Buszko [2], Janusz Pawliszyn [3], Markus Selzner [4,5] and Barbara Bojko [1,*]

[1] Department of Pharmacodynamics and Molecular Pharmacology, Faculty of Pharmacy, Nicolaus Copernicus University in Torun, Collegium Medicum in Bydgoszcz, 85-089 Bydgoszcz, Poland; i.stryjak@cm.umk.pl (I.S.); n.warmuzinska@cm.umk.pl (N.W.); k.luczykowski@cm.umk.pl (K.Ł.); patrycja.janiszek@gmail.com (P.J.)

[2] Department of Biostatistics and Biomedical Systems Theory, Faculty of Pharmacy, Nicolaus Copernicus University in Torun, Collegium Medicum in Bydgoszcz, 85-089 Bydgoszcz, Poland; kacper.wnuk@cm.umk.pl (K.W.); buszko@cm.umk.pl (K.B.)

[3] Department of Chemistry, University of Waterloo, Waterloo, ON N2L 3G1, Canada; hrosales@uwaterloo.ca (H.R.-S.); janusz@uwaterloo.ca (J.P.)

[4] Ajmera Transplant Center, Department of Surgery, Toronto General Hospital, University Health Network, Toronto, ON M5G 2N2, Canada; peter.urbanellis@queensu.ca (P.U.); markus.selzner@uhn.ca (M.S.)

[5] Department of Medicine, Toronto General Hospital, Toronto, ON M5G 2C4, Canada

[*] Correspondence: bbojko@cm.umk.pl

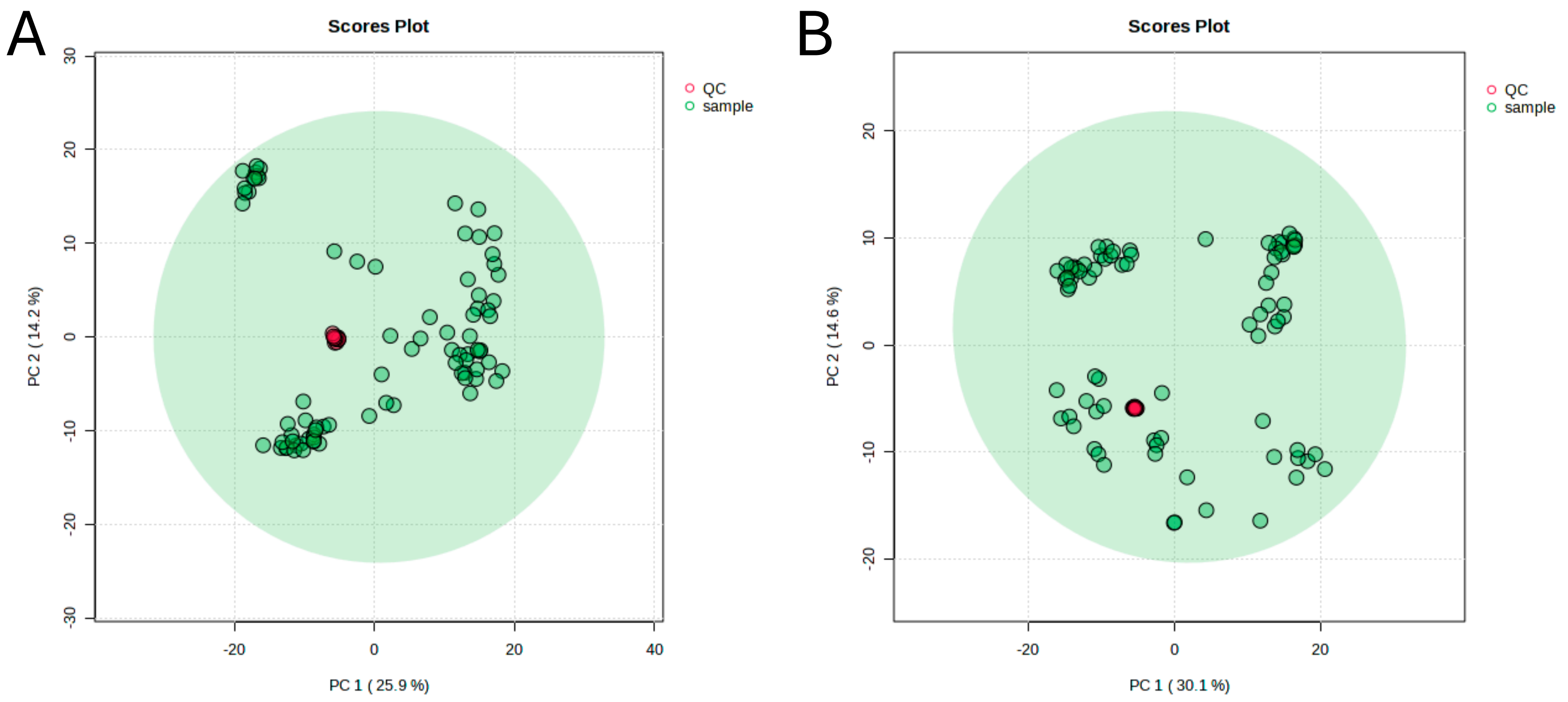

Figure S1 Principal component analysis (PCA) plots of all analyzed samples and extraction quality control samples (red). A – positive ionization mode; B – negative ionization mode

Table S1. The list of metabolites showed statistically significant changes during NEVLP, HMP and SCS kidney preservation.

Metabolite	Molecular Weight	RT [min]	Slope	FDR adjusted p-value	Adjusted R2	Ionization mode
**NEVKP**						
Pantothenate	219.10958	7.27	-0.2613952	0.00556506	0.84833201	negative
Dehydroisoandrosterone 3-glucuronide	464.2403	13.27	0.29396545	0.02702422	0.69774962	negative
Histidinyl-Histidine	292.1279	6.07	-0.2351426	0.0190418	0.74465375	negative
Tocopheronic acid	294.14616	14.50	0.25427636	0.01654286	0.7636466	negative
Indole	117.05804	9.52	0.14878183	0.01252482	0.78205762	positive
N-acetyl- Alanine<br>2-oxo-5-amino-pentanoic acid<br>Propionylglycine<br>3-Hydroxyproline<br>cis-4-Hydroxyproline<br>5-Aminolevulinic acid<br>N-Acetyl-beta-alanine	131.05828	1.45	-0.1870806	0.01252482	0.77998332	positive
4-Imidazolone-5-propanoic acid<br>5-Hydroxymethyl-4-methyluracil<br>Imidazolelactic acid	156.05345	3.73	0.15971601	0.04288184	0.69571169	Positive
Methionine sulfoxide	165.04591	1.645	0.09950459	0.02593835	0.73244786	positive
Phenylalanine	165.07883	9.515	0.13183092	0.01224925	0.78746472	positive
Indole-3-propionic acid<br>Indole-3-methyl acetate	189.07879	9.508	0.12576163	0.00764656	0.82587929	positive
Leucyl-Alanine<br>Isoleucyl-Alanine<br>Alanyl-Leucine<br>Alanyl-Isoleucine	202.13169	8.262	0.08890131	0.02029235	0.74882833	positive
Pantothenate	219.11054	7.279	-0.253836	0.02029235	0.74967001	positive
Formylkynurenine	236.07948	12.246	0.03661907	0.02029235	0.74929876	positive
**HMP**						

epsilon-Tocopherol<br>gamma-tocotrienol	410.31752	23.981	-0.0918403	0.03768108	0.66878867	negative
5-Acetylamino-6-formylamino-3-methyluracil	226.07101	12.185	0.03306356	0.01553165	0.77182683	negative
Cystine	240.02302	1.398	0.236525	0.03768108	0.66863593	negative
Pyridine N-oxide glucuronide	272.07662	12.186	0.03014962	0.01654286	0.76079661	negative
**SCS**						
Lysophosphatidic acid(8:0)	298.11927	1.967	0.16650328	0.03290391	0.68063961	negative

Table S2. The list of metabolites showed statistically significant changes prior to organ harvesting and after reperfusion and POD3

**Metabolite**	**Molecular Weight**	**RT [min]**	**SCS donor vs reperf**	**SCS donor vs POD3**	**NEVKP donor vs reperf**	**NEVKP donor vs POD3**	**HMP donor vs reperf**	**Ionization mode**
Erythronic acid<br>Threonic acid	136.03687	1.68	-0.27133	-0.89558	-1.2547	-1.7476	-1.6587*	negative
6-Hydroxypseudooxynicotine	194.10597	14.19	-1.6911	-1.5938	-1.3846	2.8795	-2.1166*	negative
Gluconic acid<br>Galactonic acid<br>Gulonic acid	196.05793	1.26	-0.44845	-1.1487	-1.0116	-0.81237	4.006*	negative
N-Acetyl-S-(N-methylcarbamoyl)cysteine	220.05156	1.25	2.6878	-0.27092	2.0071	-0.66812	3.3671*	negative
5-Acetylamino-6-formylamino-3-methyluracil	226.06919	1.68	0.22098	-0.36227	-0.15278	-0.48608	-1.3605*	negative
Thymidine	242.08963	1.73	-0.529	0.63913	0.60408	0.12433	-0.37038*	negative
Pseudouridine	244.06872	1.68	-0.79446	1.0633	-1.6633	0.68919	-1.4678*	negative
5,6-Dihydrouridine<br>Aspartyl-hydroxyproline	246.08436	1.68	0.20788	1.8166	-1.1408	1.5745	-0.86587*	negative

Ascorbic acid-2-sulfate	255.98821	1.61	0.23867	4.0749	-0.51904	3.2896*	-0.35213*	negative
3-Methyluridine<br>5-Methyluridine<br>Imidazoleacetic acid riboside	258.0847	1.69	-0.14155	1.5632	-1.5285	-0.2957	-0.87729*	negative
1-Methylinosine	282.09584	1.70	-2.2269	0.065714	-1.5211	-0.77018	-2.0217*	negative
13Z-octadecenoic acid<br>7Z-octadecenoic acid<br>Octadec-9-enoic Acid<br>Oleic acid	282.25516	21.82	0.19686	2.0944	-0.046721	-0.21981	-2.6505*	negative
Guanosine	283.091	1.68	-2.1537	-2.04	-1.6648	-1.2928	-1.9777*	negative
Xanthosine	284.07658	12.81	1.4513	0.3349	1.8705	-0.87766	2.6189*	negative
p-Cresol glucuronide	284.08898	9.14	-0.1625	-0.69305	1.5359	-1.3963	-1.6578*	negative
Stearic acid	284.27076	22.56	0.070775	-0.80663	-1.5788	-1.5132	-2.1663*	negative
hydroxy stearic acid<br>3R-hydroxy-octadecanoic acid	300.26582	21.32	-0.23378	-0.43256	-1.3328	-1.6343	-2.1955*	negative
Cytidine 2',3'-cyclic phosphate<br>Deoxycytidine monophosphate	305.0409	1.68	-0.67662	1.2394	-1.4178	1.1333	-1.7105*	negative<br>negative
Deoxyuridine monophosphate	308.04143	1.68	0.93791	-0.068051	-0.57264	-1.237	-2.4176*	negative
Indoxyl glucuronide	309.08424	9.11	-0.087626	4.3624	-1.1323	4.65*	-0.82058	negative
N-Acetylneuraminic acid	309.10537	1.25	-2.0466	-0.72944	-1.0671	1.1401	-2.0738*	negative
4-Oxo-13-cis-retinoate<br>all-trans-4-oxoretinoic acid	314.18755	13.72	3.3555	2.0646	2.9782*	-0.81877	3.5756*	negative
15-deoxy-delta-12,14-PGJ2<br>6b-Hydroxymethandienone<br>7-Oxo-8,15-isopimaradien-18-oic acid<br>all-trans-18-Hydroxyretinoic acid<br>all-trans-4-hydroxyretinoic acid<br>all-trans-5,6-Epoxyretinoic acid	316.20313	17.36	-0.20013	-0.074683	-1.2987*	0.40974	-2.0381	negative

3'-UMP<br>Pseudouridine 5'-phosphate<br>Uridine 5'-monophosphate<br>Uridine 2'-phosphate	324.03509	1.38	-3.7598	-3.5519	-0.83655	-0.17941	-2.2755*	negative
(9R,10S,12Z)-9,10-Dihydroxy-8-oxo-12-octadecenoic acid	328.22409	15.15	-3.0155	-0.78298	-3.4496*	-0.5443	-1.3445*	negative
Inosine 2',3'-cyclic phosphate	330.03515	12.18	0.99336	0.33213	1.6091	0.037199	1.836*	negative
11-Dehydrocorticosterone	344.19785	14.76	5.5271	-1.0794	7.7215*	-0.82377	6.9008*	negative
8-Oxo-dGMP<br>Guanosine monophosphate<br>Cyclic pyranopterin monophosphate	363.0573	1.68	-2.9622	-3.9355	-0.64427	-0.0765	-2.2756*	negative
DHOPA	374.20859	12.68	5.8309	6.5906	6.3507*	-0.90052	5.9356*	negative
Resolvin D1<br>Resolvin D2<br>Neuroprostane	376.22412	12.40	7.4263	5.7451	8.2672*	-0.97098	7.7928*	negative
NNAL-N-glucuronide	386.15692	12.89	1.4431	4.3354	3.3049	3.9093*	2.7858*	negative
N-oleoyl phenylalanine	429.32326	21.82	-0.12069	4.9174	-1.1862	-0.89154	-1.5451*	negative
Glycochenodeoxycholic acid<br>Glycodeoxycholic acid<br>Glycoursodeoxycholic acid	449.31319	13.85	-2.4479	-0.9394	-2.2828	-1.6994	-2.2595*	negative
Glycochenodeoxycholic acid<br>Glycodeoxycholic acid<br>Glycoursodeoxycholic acid	449.31334	15.09	-1.25	-0.66367	-0.62158	-1.313	-1.4699*	negative

Dehydroepiandrosterone 3-glucuronide<br>Dehydroisoandrosterone 3-glucuronide<br>Testosterone glucuronide	464.2403	13.27	1.2483	-0.99447	3.6806	-0.85107	2.2471*	negative
Glycocholic acid	465.30784	12.54	-2.3577	0.3837	-3.9535	-1.4138	-3.1917*	negative
Creatinine	113.0589	4.28	-0.26765	3.5544	-0.69552	3.0342*	-0.29441	positive
Guanidinoacetic acid	117.054	2.2	0.11919	-1.993	-1.2665	-3.4279	-1.3361*	positive
Indole	117.0579	12.26	1.4615	0.98827	1.5429	0.39187	1.4246*	positive
Valine<br>N-Methyl-a-aminoisobutyric acid<br>5-amino-pentanoic acid	117.0792	1.73	-0.80197	-2.5684*	-1.449	-2.9395	-0.89928*	positive
Pyroglutamic acid	129.0426	1.53	-1.3987	-1.4723	-1.0026	0.032906	-1.044*	positive
Pipecolic acid	129.079	2.28	-0.74307	0.79395	0.57645	1.5334*	-1.5154	positive
2-oxo-5-amino-pentanoic acid<br>N-acetyl-Alanine<br>hydroxyproline<br>Propionylglycine<br>N-Acetyl-beta-alanine<br>5-Aminolevulinic acid<br>Glutamic gamma-semialdehyde	131.0583	1.45	-0.80791	-0.9776	-1.2532	-0.53506	-1.2478*	positive
Glutaric acid<br>Methylsuccinic acid	132.0423	1.3	-0.15115	-0.21324	-0.22912	0.11456	4.3545*	positive
Aspartic acid	133.0376	1.46	-1.5813	-0.1753	-0.28616	1.2536*	-1.5527	positive
Indoxyl	133.0529	9.1	-1.5912	3.2409	-1.4364	3.1992*	-1.9213*	positive

Adenine	135.0545	9.1	0.33646	-1.0616	-2.1184	-1.268	1.9708*	positive
Hypoxanthine	136.0384	7.44	2.1867	2.9546	0.31111	1.4336	1.1947*	positive
1,3-Dimethyluracil	140.0585	7.17	-0.87861	2.7462	1.4133	2.9184*	1.7415*	positive
3-Dehydroxycarnitine	145.1103	11.46	-2.578*	-3.9872*	-1.4312	-3.3427*	-1.1851*	positive
Alanylglycine Glutamine	146.0691	1.53	-1.5963	-2.1353	-0.9786	-0.36684	-1.4628*	positive
Lysine	146.1055	2.28	-0.74554	0.79436	0.57341	1.5351*	-1.5158	positive
N6-Methyladenine	149.0701	7.16	-0.6281	2.0829	0.46165	2.8362*	8.9*	positive
Hydroxyadenine Guanine	151.0494	5.34	0.59143	2.9507	-0.0003897	0.27974	-0.90913*	positive
Histidine	155.0694	2.28	4.8243*	1.9715	4.9547*	0.84492*	4.1521*	positive
4-Imidazolone-5-propanoic acid Imidazolelactic acid	156.0535	3.73	1.4181	1.4488	4.8614*	1.2293	2.3945*	positive
N-Acetylproline	157.0735	2.27	5.0292*	2.0707	5.0369*	0.69314	4.3962*	positive
2-Aminoadipic acid	161.0688	1.9	1.7951	4.3648	1.7054	3.88*	1.8242*	positive
Carnitine	161.1051	4.96	-0.14558	2.0212	-1.5502	1.3152	-1.2232*	positive
Fucose 1,5-Anhydrosorbitol	164.0685	1.28	4.2691	1.099	2.6213	-0.91376	4.8018*	positive
N2-Methylguanine	165.065	7.55	-0.08022	2.1925	-0.6689	1.5658*	-0.1139	positive
Uric acid	168.0283	1.77	-0.20871	2.8554	0.10427	2.1705*	0.19238	positive
N-Acetyl-glutamate 5-semialdehyde	173.0687	1.76	-0.0027637	-0.022468	-0.91055	0.9679	-0.94663*	positive
Indoleacetic acid	175.0633	11.27	1.508	1.9426	-0.18206	-2.8359*	0.58924	positive

5-Hydroxyindoleacetaldehyde<br>2-Keto-3-deoxy-gluconic acid<br>3-Keto-b-galactose<br>Gulonolactone<br>Gluconolactone<br>Arabino-hexos-2-ulose<br>Galactonolactone	178.0476	1.3	-0.23634	0.34075	-0.45697	0.58306	6.4432*	positive
2-Amino-3,4-dihydroxypentanedioic acid<br>Isoxanthopterin	179.0439	10.89	-0.66325	-2.5228*	-1.0781	-2.8761	-1.6852	positive
Hippuric acid	179.0581	9.12	-1.0328	0.80346	-1.8615	-0.42507	-1.306*	positive
Salsolinol	179.0945	9.16	-1.3985	-0.62428	0.42061	1.5415	2.1279*	positive
5-Methylthioribose	180.0455	1.76	-0.46392	2.8482	-0.0083314	3.512*	-0.23756	positive
Galactitol<br>Sorbitol	182.0788	1.28	3.5898	-0.21053	4.2998*	1.1666	5.7819*	positive
Indoleacrylic acid	187.0631	12.26	1.4347	0.9792	1.5108	0.44254	1.3926*	positive
Kynurenic acid	189.0426	18.45	0.10701	-0.22567	1.6725*	0.1267	-0.39546	positive
5-Hydroxyindoleacetic acid	191.058	10.15	0.0058181	1.8546	0.45785	2.0474	1.0555*	positive
Gluconic acid<br>Galactonic acid<br>Gulonic acid	196.0582	1.3	-0.55008	-0.16925	-0.094066	0.23932	5.2241*	positive
O-Phosphohomoserine<br>O-Phosphorylhomoserine<br>Iminoerythrose 4-phosphate<br>O-Phosphothreonine	199.0256	1.27	1.1163	0.28585	-0.18657	-1.5617	2.0731*	positive

Leucyl-Alanine<br>Alanyl-Isoleucine<br>Isoleucyl-Alanine<br>N-Acetylisoputreanine<br>Alanyl-Leucine	202.1317	8.26	0.70768	4.2009*	0.27008	3.7933*	0.63485	positive
Glycyl-Lysine<br>Lysyl-Glycine	203.127	8.64	-0.52331	6.0089	-0.43182	4.8797*	0.13194	positive
Tryptophan	203.127	12.25	1.4372	0.97896	1.5109	0.44339	1.3919*	positive
Kynurenine<br>Formyl-5-hydroxykynurenamine	208.0848	10.15	-0.024518	1.8114	0.57558	2.1329	0.9254*	positive
Hydroxyphenylacetylglycine	209.0687	7.93	-0.37771	6.3176*	-0.20855	6.0772*	-0.44421	positive
O-Phospho-4-hydroxy-threonine	215.0204	1.28	-0.17894	0.046716	-0.26777	0.59176	3.8182*	positive
Propenoylcarnitine	215.1156	9.31	-0.33712	4.0019	-0.19774	4.612*	-0.35974	positive
5-Hydroxy-tryptophan	220.0846	12.26	1.376	0.95876	1.5291	0.55472	1.4735*	positive
Hydroxykynurenine	224.0796	20.99	-2.0935	-5.6013	-2.8922*	-8.8505*	-3.2168*	positive
Prolylhydroxyproline<br>Hydroxyprolyl-Proline<br>Pyroglutamylvaline	228.1109	7.29	-0.28127	0.81186	0.065019	1.206*	0.19632	positive
Isoleucylproline	228.1472	9.59	1.068*	5.4105*	0.90373	4.8148*	1.5809*	positive
Leucylproline	228.1473	9.11	-0.044379	3.7634	-1.7689	3.766*	-0.43598	positive
Isobutyryl-carnitine<br>O-butanoyl-carnitine	231.1469	15.36	1.6472	3.5688	-0.60789	2.6715	1.2552*	positive
Formylkynurenine	236.0795	12.25	2.1701	1.494	1.6537	0.24306	2.4177*	positive
Uridine	244.0695	1.78	-0.11945	2.6842*	-0.66338	2.3181*	-0.41361	positive
cyclic 6-Hydroxymelatonin<br>N-acetyltryptophan	246.1001	11.34	0.7074	2.2934	7.8561*	3.396*	0.93781	positive
Neopterin	253.0822	9.12	-0.044111	0.030274	-0.51197	0.042484	6.2639*	positive
1-(beta- Ribofuranosyl)-1,4-dihydronicotinamide	256.1056	1.8	3.8004	2.7232	0.63259	2.759*	2.8732*	positive

Glycerylphosphorylcholine	257.1026	1.39	-2.1214	-2.1589	-1.651	-0.38796	-1.7767*	positive
N-Lauroylglycine	257.1989	14.62	-1.1805*	-0.17423	0.1126	0.80004	0.1605	positive
hydroxyisovaleroyl carnitine	261.1581	22.27	-1.756	-1.8103	-1.0382	-2.6451*	-3.436	positive
Neuraminic acid<br>Deoxyguanosine<br>Adenosine	267.0965	7.69	-4.0378*	-2.3536	-2.3327	-1.4672	0.23623	positive
Inosine	268.0805	1.78	-2.2307	-2.3627	-1.1605	-0.92468	-1.7814*	positive
19-Norandrosterone	276.2087	27.64	0.072831	0.74721	-0.4799	-1.0123	-2.3567*	positive
DOPA sulfate	277.0248	1.27	1.711	0.41881	1.4507	0.020846	2.8796*	positive
trans-vaccenic acid	282.2554	21.92	0.59369	1.384	0.73709	0.56006	-1.3373*	positive
Epitestosterone<br>Dehydroepiandrosterone<br>5beta-androstane-3,17-dione<br>5alpha-androstane-3,17-dione<br>Dehydroandrosterone<br>Testosterone	288.2088	12.17	-0.73825	4.9626	-0.78416	5.2098*	0.04182	positive
5alpha-dihydrotesosterone<br>Epiandrosterone<br>Androstenediol<br>3alpha-hydroxy-5beta-androstan-17-one<br>5beta-Dihydrotestosterone<br>Epietiocholanolone<br>4-Androstenediol<br>5beta-dihydroepitestosterone<br>Androsterone	290.2239	19.85	-1.2665	-0.90385	-0.51311	-1.5423	-2.7853*	positive
10Z-nonadecenoic acid	296.2714	23.83	0.24761	0.46938	-0.80236	-1.8085	-4.9364*	positive
4-Oxoretinal<br>All-Trans-3,4-Didehydro-Retinoic acid<br>Norethindrone	298.193	15.91	0.0027732	0.49906	-0.4575	3.969*	-1.5108*	positive

9-cis-retinoic acid<br>4-Oxoretinol<br>4-OH-Retinal<br>Retinoic Acid<br>3-hydroxynonanoyl carnitine	300.2088	11.42	-0.86881	3.032	1.8693	2.9999*	2.2734*	positive
Leukotriene A4	317.2208	24.13	-0.89713	-2.13	-0.64674	-2.8518*	-4.7589	positive
11R-HEPE<br>15S-HEPE<br>5S-HEPE<br>18R-HEPE<br>15R-HEPE<br>Galactosylhydroxylysine	318.2192	11.42	-0.51872	3.0709	1.7755	2.9799*	2.059*	positive
11-deoxycorticosterone<br>6-beta-hydroxyprogesterone<br>17alpha-hydroxyprogesterone<br>6(beta)-hydroxyprogesterone<br>11-Hydroxy-delta-9-THC<br>8-Hydroxy-delta-9-THC<br>11-hydroxy-Delta(9)-tetrahydrocannabinol<br>7-beta-Hydroxy-delta-9-THC<br>8-beta-Hydroxy-delta-9-THC	330.2191	11.42	-0.6289	3.3478	2.2368	3.4504*	2.2838*	positive
N-oleoyl glycine	336.2661	26.11	0.79826	-0.2791	0.026451	-0.14311	-4.5191*	positive
19-Oxo-deoxycorticosterone<br>11-nor-9-carboxy-Delta(9)-tetrahydrocannabinol<br>11-Dehydrocorticosterone<br>7'-Carboxy-alpha-tocotrienol	344.1985	13.81	-1.4823	-3.7377	-1.9873	-2.5073*	-0.74865	positive

21-Deoxycortisol<br>corticosterone<br>11-deoxycortisol<br>19-Hydroxydeoxycorticosterone<br>12-Hydroxy-11-methoxy-8,11,13-abietatrien-20-oic acid<br>21-Hydroxy-5b-pregnane-3,11,20-trione	346.2142	14.15	-3.1335*	-3.6894*	-0.51761	-3.1161*	-1.8807*	positive
7'-Carboxy-alpha-chromanol<br>3b,15b,17a-Trihydroxy-pregnenone<br>3beta,17alpha,21-Trihydroxy-pregnenone<br>11b,21-Dihydroxy-5b-pregnane-3,20-dione<br>3a,21-Dihydroxy-5b-pregnane-11,20-dione	348.2297	11.43	-0.61876	3.3442	2.2542	3.4416*	2.1879*	positive
(5Z)-(15S)-11alpha-Hydroxy-9,15-dioxoprostanoate	352.2248	12.5	-5.0532*	-1.2641	3.8069	-0.45391	-4.2459	positive
Aldosterone	358.214	12.44	7.7597*	6.4992	8.7129*	-0.050874	8.3732*	positive
19-Oic-deoxycorticosterone<br>Cortisone	360.1934	12.97	-2.0524*	-2.6356	-1.6576	-1.4437	-1.4203*	positive
18-Hydroxycorticosterone	362.063	2.28	6.2583	1.9977	5.6288	0.091217	4.9959*	positive
4,5beta-Dihydrocortisone<br>11b,21-Dihydroxy-3,20-oxo-5b-pregnan-18-al	362.2089	12.03	-1.0228	-0.56463	-0.87771	-1.1985	-1.4532*	positive
Cortisol<br>18-Hydroxycorticosterone<br>4,5beta-Dihydrocortisone<br>11b,21-Dihydroxy-3,20-oxo-5b-pregnan-18-al	362.209	12.51	-2.2565	-2.5296	0.93618*	-1.116	0.35203	positive
DHOPA	372.1935	12.99	3.7426*	3.7342	6.1728*	1.3103	5.5159*	positive

Resolvin D1<br>Resolvin D2<br>11beta-Hydroxy-3,20-dioxopregn-4-en-21-oic acid<br>4,6-cholestadienone	376.2246	12.45	8.0444*	6.7769	8.9999*	-0.081578	8.6952*	positive
3beta-Hydroxy-5-cholestenal<br>27alpha-Hydroxy-8-dehydrocholesterol<br>5,6-trans-25-Hydroxyvitamin D3<br>7-Ketocholesterol<br>25-Hydroxytachysterol3<br>(3beta,5alpha,6a)-Cholesta-8,14-diene-3,6-diol<br>7alpha-Hydroxy-4-cholesten-3-one<br>1alpha-hydroxyvitamin D3 / 1alpha-hydroxycholecalciferol<br>25-hydroxyvitamin D3 / 25-hydroxycholecalciferol / calcidiol	400.3339	23.96	0.99575	-1.8373	-0.45352	-2.6056*	-2.694	positive
22-Dehydroclerosterol<br>5-Dehydroavenasterol	410.3546	25.43	-0.56107	-2.5412	-0.32612	-2.5195	-3.1851*	positive
N-Docosahexaenoyl GABA	410.3909	25.04	0.044349	-0.51507	-0.55071	1.6497*	-5.3978*	positive
(24R)-24,25-Dihydroxycalciol	413.2927	13.9	-3.027	-0.18502	-1.8268	-0.84596	-2.5724*	positive

25,26-dihydroxyvitamin D<br>24R,25-Dihydroxyvitamin D3<br>7 alpha,24-Dihydroxy-4-cholesten-3-one<br>7 alpha,26-Dihydroxy-4-cholesten-3-one<br>7alpha,25-dihydroxycholestenone<br>23S,25-dihydroxyvitamin D3<br>3 beta-Hydroxy-5-cholestenoate<br>1alpha,25-dihydroxyvitamin D3 / 1alpha,25-dihydroxycholecalciferol / calcitriol<br>24,25-Dihydroxyvitamin D<br>7alpha,24-dihydroxycholest-4-en-3-one<br>(25R)-7alpha,26-dihydroxycholest-4-en-3-one	416.3287	22.12	0.40402	0.27623	-1.8807	-1.2973	-1.1131*	positive
Glycohyocholic Acid<br>Glycocholic acid	465.3089	13.3	-3.6205*	-0.73224	-2.1695	-0.18326	-2.7774*	positive
Ursodeoxycholic acid 3-sulfate<br>Chenodeoxycholic acid sulfate	472.2518	9.34	-0.16021	4.0042	1.2492	4.5898*	2.1292	positive
LPE(18:1)	479.301	18.01	0.89428	0.76898	-0.53687	-0.17114	-1.1483*	positive
2'-Deoxyinosine triphosphate	491.9856	9.94	-1.9784	-0.52253	-1.2008*	-0.32043	-1.2923*	positive

For clarity, phospholipids were removed from the Table; lipidomics analysis is presented in Warmuzińska et al. Front Mol Biosci 2024, 11, 1341108
